# Lithia-Based Nanocomposites Activated by Li_2_RuO_3_ for New Cathode Materials Rooted in the Oxygen Redox Reaction

**DOI:** 10.1186/s11671-019-3223-4

**Published:** 2019-12-16

**Authors:** Byeong Gwan Lee, Yong Joon Park

**Affiliations:** Department of Advanced Materials Engineering, Kyonggi University, 154-42, Gwanggyosan-Ro, Yeongtong-Gu, Suwon-Si, Gyeonggi-Do 16227 Republic of Korea

**Keywords:** Anionic redox, Cathode, Lithia, Nanocomposite, Lithium battery

## Abstract

Lithia-based materials are promising cathodes based on an anionic (oxygen) redox reaction for lithium ion batteries due to their high capacity and stable cyclic performance. In this study, the properties of a lithia-based cathode activated by Li_2_RuO_3_ were characterized. Ru-based oxides are expected to act as good catalysts because they can play a role in stabilizing the anion redox reaction. Their high electronic conductivity is also attractive because it can compensate for the low conductivity of lithia. The lithia/Li_2_RuO_3_ nanocomposites show stable cyclic performance until a capacity limit of 500 mAh g^−1^ is reached, which is below the theoretical capacity (897 mAh g^−1^) but superior to other lithia-based cathodes. In the XPS analysis, while the Ru 3d peaks in the spectra barely changed, peroxo-like (O_2_)^n−^ species reversibly formed and dissociated during cycling. This clearly confirms that the capacity of the lithia/Li_2_RuO_3_ nanocomposites can mostly be attributed to the anionic (oxygen) redox reaction.

## Introduction

Our society is becoming increasingly more reliant on energy storage systems (ESSs) due to greater use of cell phones, laptops, and electric vehicles. In addition, electricity generated from eco-friendly power generation systems needs to be stored in large energy storage systems (ESSs). These examples of ESSs are mostly based on secondary battery systems, which has led to rapid growth of the market share of Li-ion batteries (LIBs), considered the most advanced secondary battery. However, the energy density of current LIBs is not sufficient to meet the requirements of many applications [1–7]. Hence, much research has focused on improvement of the energy density of battery systems. In particular, development of a superior cathode material exhibiting higher reversible capacity than conventional transition metal-based oxides is of great research interest [1–7].

Several materials compatible with the anion (oxygen) redox reaction may hold promise for cathodes with high energy density [8–16]. The reversible discharge capacity of cathode materials currently used is based on the redox reaction of cationic transition metal ions in the compounds. However, introduction of an anionic redox reaction rooted in oxygen could potentially drive high reversible capacity, overcoming the capacity limits of transition metal oxides. For example, the oxygen redox reaction (ORR) is mainly responsible for the high energy density of Li-rich materials, such as *x*Li_2_MnO_3_ − (1 − *x*)Li(Ni,Mn)O_2_ [17, 18]. When Li-rich materials are charged over the cation (transition metal oxide) redox reaction region, the reversible ORR (2O^2−^ ➔ O_2_^*x*−^) progresses. This reaction contributes to the reversible capacity of Li-rich materials in conjunction with the cation redox reaction of transition metal ions. However, these reactions require a high charge voltage (> 4.5 V) in order to activate the ORR, which drives degradation of the organic electrolyte and causes serious capacity fading [19–23].

Lithia (Li_2_O)-based compounds have recently been suggested as cathode materials based on the ORR [24–29]. While the reversible capacity of Li-rich compounds is mainly attributed to the cation redox reaction of the transition metal ions, that of the lithia-based compounds is nearly purely dependent upon the anion (oxygen) redox reaction between O^2−^ and O^*x*−^ (0.5 ≤ *x* < 2). This battery chemistry is comparable with Li-air batteries, in that it mainly uses the ORR. However, the fundamental redox reaction of the Li-air battery is based on the phase transition from gas (O_2_) to solid (Li_2_O_2_); this reaction is not solely a “phase transition” without composition change, but a chemical reaction involving Li-ions. However, it is referred to as a “phase transition” process in this field of research because it is accompanied by a phase change. Capacity fading and high overpotential of the Li-air cells occur due to instability of the contact between gas and solid, resulting in slow reaction kinetics [30–36]. In contrast, oxygen ions in lithia-based compounds maintain the solid phase without a phase transition during charging and discharging processes. Therefore, lithia-based compounds can be classified as a new cathode material for LIBs rather than a subclass of Li-air electrodes.

**Scheme 1:**
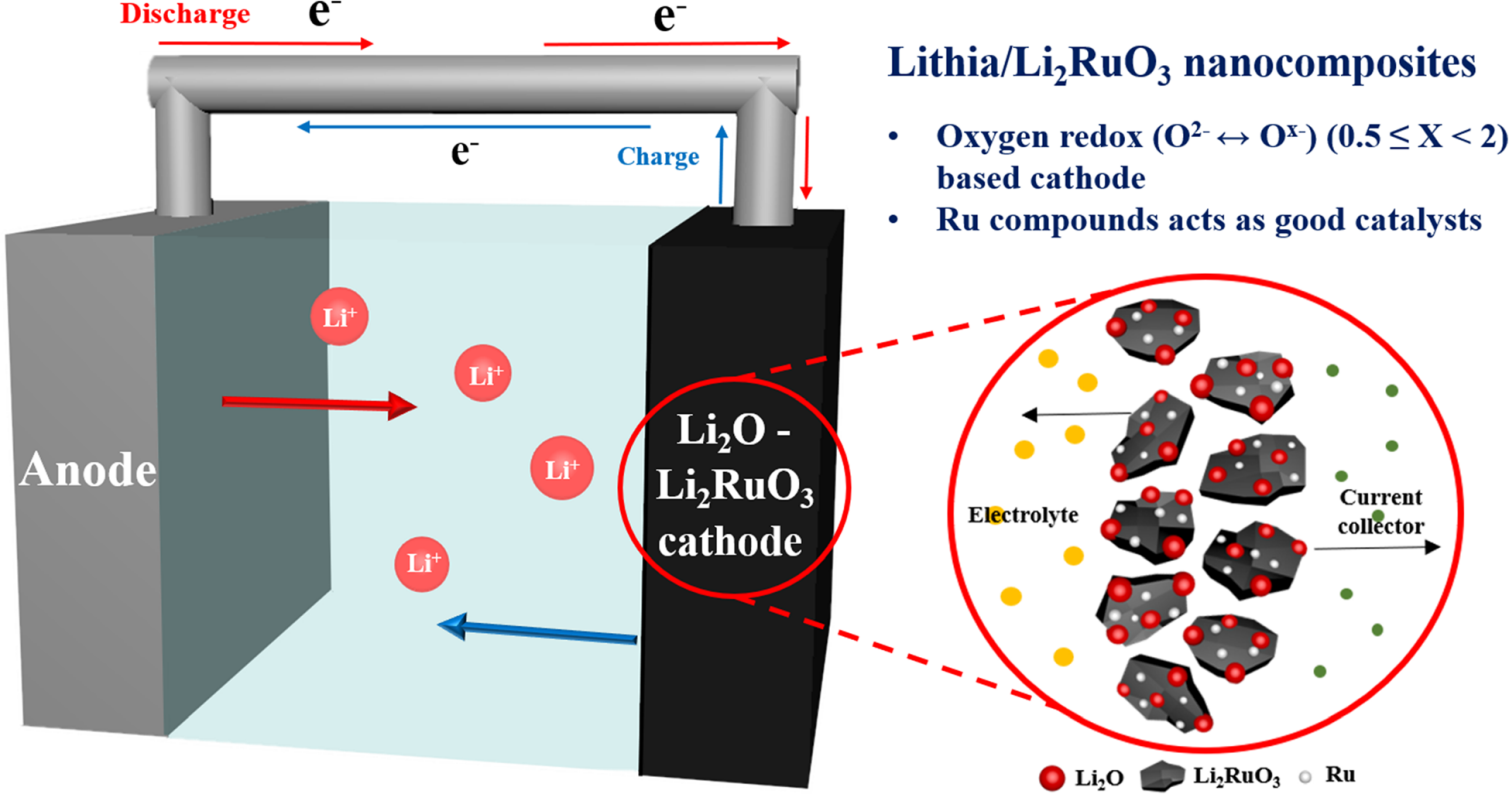
Schematic diagram showing the structure of the lithia/Li_2_RuO_3_ nanocomposites and essential concepts in this study.

Actually, oxygen ions in solid lithia are hardly activated during the charging process. Therefore, catalysts (sometimes referred to as the dopant) are essential for activating oxygen ions in lithia and stabilizing the reaction products (Li_2_O_2_ or LiO_2_). Co, Fe, and Cu oxides have been used as catalysts for activating lithia [24–29], whereby the electrochemical performance of lithia-based compounds is extremely sensitive to the composition and amount of catalyst present. As part of our efforts to explore a more efficient catalyst, Li_2_RuO_3_ is introduced as a new catalyst for the activation of lithia in this study. We noted that Ru ion plays a role in stabilizing the anion redox reaction in Li-rich oxides [37–39], and that Ru-based Li-rich oxides exhibit more reversible oxygen redox processes and are structurally more stable against oxygen release compared to Co, Ni, and Mn-based Li-rich oxides. This implies that Ru ion has the potential to be a more stable and better catalyst to activate the ORR than other previously used transition metal ions (e.g., Co, Fe, and Cu). Moreover, the high electronic conductivity of Ru oxides may compensate for the insufficient conductivity of lithia. In this work, we prepared lithia/Li_2_RuO_3_ nanocomposites and investigated their properties using X-ray diffractometry (XRD), X-ray photoelectron spectroscopy (XPS), and electrochemical measurements to confirm the effects of Ru oxides as catalysts. Scheme 1 illustrates the structure of the lithia/Li_2_RuO_3_ nanocomposites and essential concepts in this study.

## Methods

Li_2_RuO_3_ was employed as the catalyst for activating nano-lithia. To form Li_2_RuO_3_, RuO_2_ (Alfa Aesar, 99.9%) and Li_2_CO_3_ (Aldrich, 99.99%) were pelletized in a 1:1 (mol%) ratio and calcined at 950 °C for 24 h in air. The calcined pellets were then pulverized into a powder. Li_2_RuO_3_ and nano-lithia (Li_2_O) powder (Alfa Aesar, 99.5%) were combined to obtain a Ru content (*f*_Ru_ = Ru/(Ru + Li)) of 0.09, then dispersed in butanol (Aldrich, anhydrous, 99.8%). The mixture was ultrasonically treated for 30 min and then filtered. The obtained Li_2_RuO_3_/Li_2_O powder was dried under vacuum at 90 °C for 24 h and then ball milled using a Planetary Mono Mill (PULVERISETTE 6, FRITSCH) to obtain the lithia/Li_2_RuO_3_ nanocomposite. Milling was performed for 150 h (resting for 30 min after milling for 1 h) at 600 rpm. Zirconia balls with 5 mm and 10 mm diameters were used in a 1:1 (wt%) ratio. The milling process was carried out under an Ar atmosphere using a glove box and sealed zirconia container. XRD patterns of the synthesized lithia/Li_2_RuO_3_ nanocomposite powder were obtained using a Rigaku Miniflex II X-ray diffractometer over a 2θ range of 10–90° with monochromatized Cu K_α_ radiation (*λ* = 1.5406 Å). To observe the degree of dispersion of Li_2_RuO_3_ and lithia in the lithia/Li_2_RuO_3_ nanocomposite, high-resolution transmission electron microscopy (HR-TEM; JEM-2100F) and energy dispersive X-ray spectroscopy (EDS) were employed.

For electrochemical tests, the positive electrode was prepared by mixing the active material (lithia/Li_2_RuO_3_ nanocomposite), carbon nanotubes, and polyvinylidene fluoride (PVDF) binder in a ratio of 60:30:10 (wt%). As a reference, a Li_2_RuO_3_ electrode was also prepared in a ratio of 80:12:8 (wt% of Li_2_RuO_3_/carbon nanotubes/PVDF). Ball milling of the electrode components was performed for 90 min. Afterward, the lithia/Li_2_RuO_3_ nanocomposite and Li_2_RuO_3_ slurry were cast on aluminum foil and dried under vacuum at 80 °C for 24 h.

Coin cells (2032-type) were used for the electrochemical tests with Li metal as the anode, 1 M LiPF_6_ in ethylene carbonate and dimethyl carbonate (1:1 v/v) containing 5 vol% vinylene carbonate as the electrolyte, and polypropylene (Celgard 2400) as the separator. The cells were assembled in an Ar-filled glove box. The lithia/Li_2_RuO_3_ nanocomposite cells were cycled through the potential range of 1.8–4.35 V with various current densities (10, 30, 100, and 200 mA g^−1^). The capacity of the cathode, calculated based on the mass of lithia, was limited to 300–600 mAh g^−1^. Li_2_RuO_3_ cells were also cycled in a potential range of 2.0–4.6 V with a current density of 30 mA g^−1^.

## Results and discussion

**Fig. 1:**
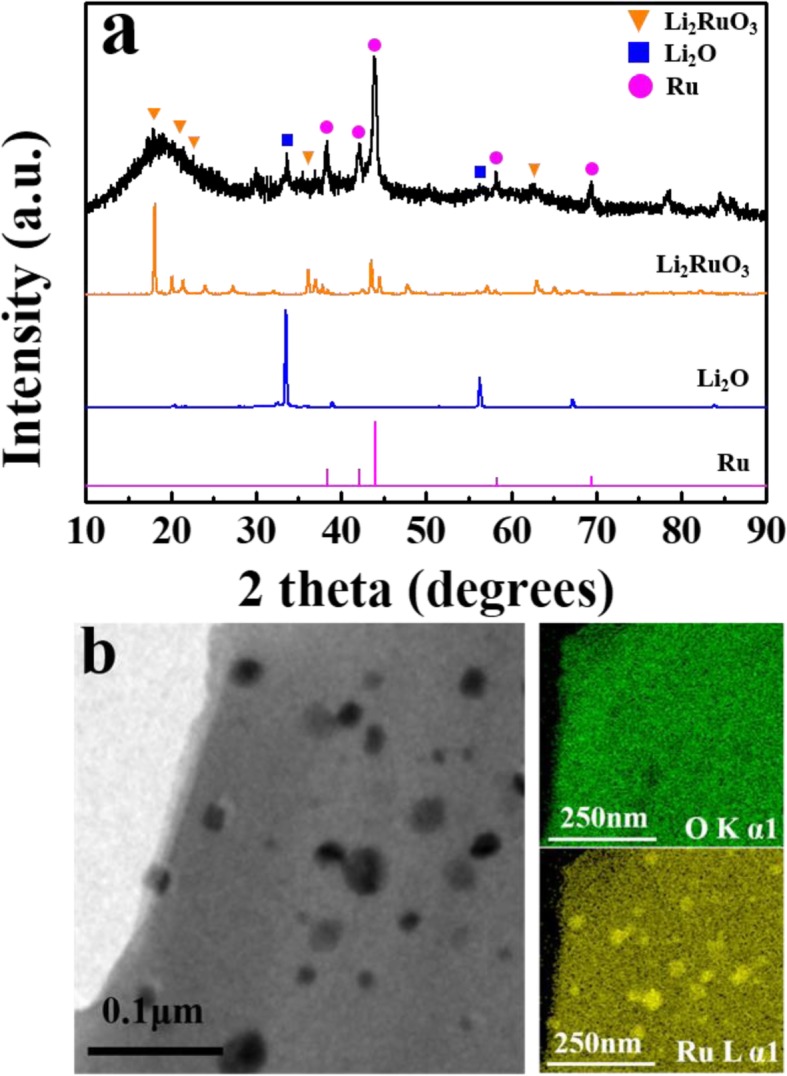
**a** XRD patterns of the lithia/Li_2_RuO_3_ nanocomposite compared with Li_2_RuO_3_, Li_2_O, and Ru. **b** TEM images and EDS element mapping of the lithia/Li_2_RuO_3_ nanocomposite powders

The structural properties of the lithia/Li_2_RuO_3_ nanocomposites were investigated using XRD and TEM. Figure 1 shows the XRD pattern of the lithia/Li_2_RuO_3_ nanocomposite, Li_2_RuO_3_, Li_2_O (lithia), and Ru. The diffraction pattern of the lithia/Li_2_RuO_3_ nanocomposite was present along with those of the raw materials (Li_2_RuO_3_ and lithia) and newly formed Ru peaks. The sharp and large Ru peaks indicate that some of the Li_2_RuO_3_ decomposed to Ru during the milling process. However, the broad but large diffraction peaks for Li_2_RuO_3_ indicate that a considerable amount of Li_2_RuO_3_ still remains, although most of the crystalline Li_2_RuO_3_ seems to have changed to an amorphous phase through the milling process. A reduction in the lithia peaks shows that crystalline lithia is also changed to an amorphous phase. The presence of an amorphous phase may result in negative effects, such as reduced electrical conductivity. However, this can also lower the overpotential because the phase transition of amorphous lithia requires less energy than that of crystalline lithia during the charge-discharge process [29].

Based on the XRD analysis, it is expected that not only amorphous Li_2_RuO_3_ but also Ru can act as catalysts for activating lithia. The distribution of catalysts and morphology of the nanocomposites were observed by HR-TEM and EDS elemental mapping. As shown in Fig. 1b, the HR-TEM image of the nanocomposite contains several dark spots, which are likely Ru particles, as supported by EDS mapping that shows a large amount of Ru in this section. Once Ru is formed by decomposition of Li_2_RuO_3_, it appears to be difficult to finely disperse in the milling process due to the ductility of metallic Ru. Therefore, some of the Ru particles appear to be agglomerated. The remaining nanocomposite material appears to be composed of homogeneously distributed lithia and catalyst, as indicated by EDS mapping.

**Fig. 2:**
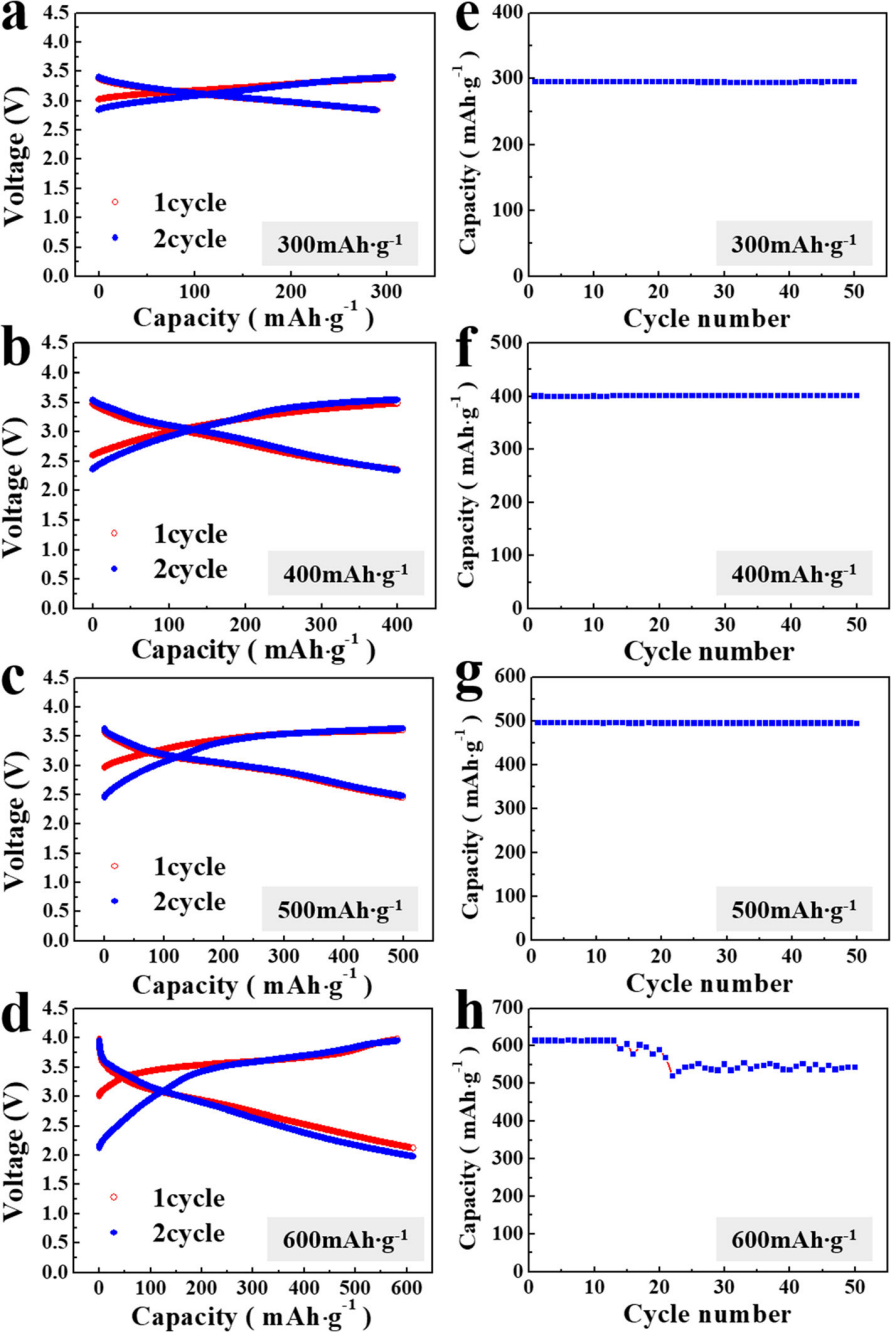
Voltage curves of the lithia/Li_2_RuO_3_ nanocomposites when the capacity is limited to **a** 300, **b** 400, **c** 500, and **d** 600 mAh g^−1^; cycling when capacity limited is corresponding to **e**, **f**, **g**, and **h**, respectively

The electrochemical properties of the cells containing lithia/Li_2_RuO_3_ nanocomposites were examined to investigate the effect of the Li_2_RuO_3_ catalyst. Figure 2 shows the voltage curves and cyclic performance of the lithia/Li_2_RuO_3_ nanocomposites when the current density is 10 mA g^−1^. When lithia is over-charged, oxygen evolution can occur because the oxidation state of oxygen can change from − 2 (Li_2_O form) to 0 (O_2_ gas) [25–27]. To check the limit-capacity where overcharging does not occur, charging-discharging capacity was limited to 300, 400, 500, and 600 mAh g^−1^, as calculated based on the mass of lithia. As shown in Fig. 2a–d, the voltage curves of the lithia/Li_2_RuO_3_ nanocomposites appear to be stable at all limited capacities during two cycles. When the capacity was limited to 300 mAh g^−1^, a narrow voltage range (from 3.5 to 2.7 V) results. However, as the limited capacity is increased, the voltage range increases as well. When the capacity was limited to 600 mAh g^−1^, the voltage rose to ~ 4.0 V at charging, and decreased to ~ 2.0 V at discharging. Figure 2e–h presents the cyclic performance of the lithia/Li_2_RuO_3_ nanocomposites under the same conditions. The nanocomposites are stable when cycled in the limited capacities of 300–500 mAh g^−1^. However, when the capacity was increased to 600 mAh g^−1^, the capacity gradually began to decrease after 13 cycles. This indicates that a stable capacity range of the lithia/Li_2_RuO_3_ nanocomposites is below 600 mAh g^−1^; overcharging may be responsible for instability of the capacity. When lithia (Li_2_O) is charged and remains in a condensed state (solid), the oxygen in the lithia changed from O^2−^ to O^*x*−^ (0.5 ≤ *x* < 2). However, when lithia was charged above the limitation for maintaining the solid form, the oxidation state of the oxygen approaches zero, and oxygen gas can be generated. This process leads to the capacity fading during cycling. However, assuming the redox reaction from lithia (Li_2_O, O^2−^) to peroxide (Li_2_O_2,_ O^1−^), the theoretical capacity of lithia is 897mAh g^−1^ [26, 27]. If the redox reaction produces O^0.5−^ (LiO_2_), the theoretical capacity of lithia increases to 1341 mAh g^−1^ [29]. Therefore, with the observed capacity below 600 mAh g^−1^, the capacity limit of lithia in the lithia/Li_2_RuO_3_ nanocomposites is not reached.

The capacity of lithia is closely associated with catalysts because the catalyst activates the lithia and stabilizes the unstable reaction products (e.g., Li_2_O_2_ and LiO_2_). The lower available capacity of lithia/Li_2_RuO_3_ nanocomposites compared with the theoretical capacity of lithia may mean that the catalyst in the nanocomposite does not activate lithia sufficiently to extract the full capacity. Stabilization of the reaction products also significantly affects the capacity stability of the lithia-based cathode. Reaction products (e.g., Li_2_O_2_ and LiO_2_) formed from the redox reaction of lithia are highly reactive. Thus, they are likely to react with other substances such as electrolytes, and transform into other materials. Suppressing these side reactions and stabilizing the reaction products is also a function of the catalyst. As shown in Fig. 2h, capacity fading after a certain number of cycles can be associated with instability of the reaction products during cycling, i.e., 600 mAh g^−1^ may be beyond the limits of which the Ru-based catalyst in the lithia/Li_2_RuO_3_ nanocomposites can stabilize the reaction products.

Although the capacity of the lithia/Li_2_RuO_3_ nanocomposites does not reach the theoretical capacity, their stable capacity (> 500 mAh g^−1^ based on the mass of lithia) is superior compared with previously reported lithia-based cathodes prepared by a milling process (< 400 mAh g^−1^) [25–28]. This suggests that Li_2_RuO_3_ and its decomposed phase (such as Ru) effectively activate lithia and stabilize reaction products during cycling. If the catalysts are more completely dispersed with lithia, they may show better catalytic activity. Other methods, such as chemical preparation (rather than mechanical milling), should be considered due to the limitations of mechanical milling.

**Fig. 3:**
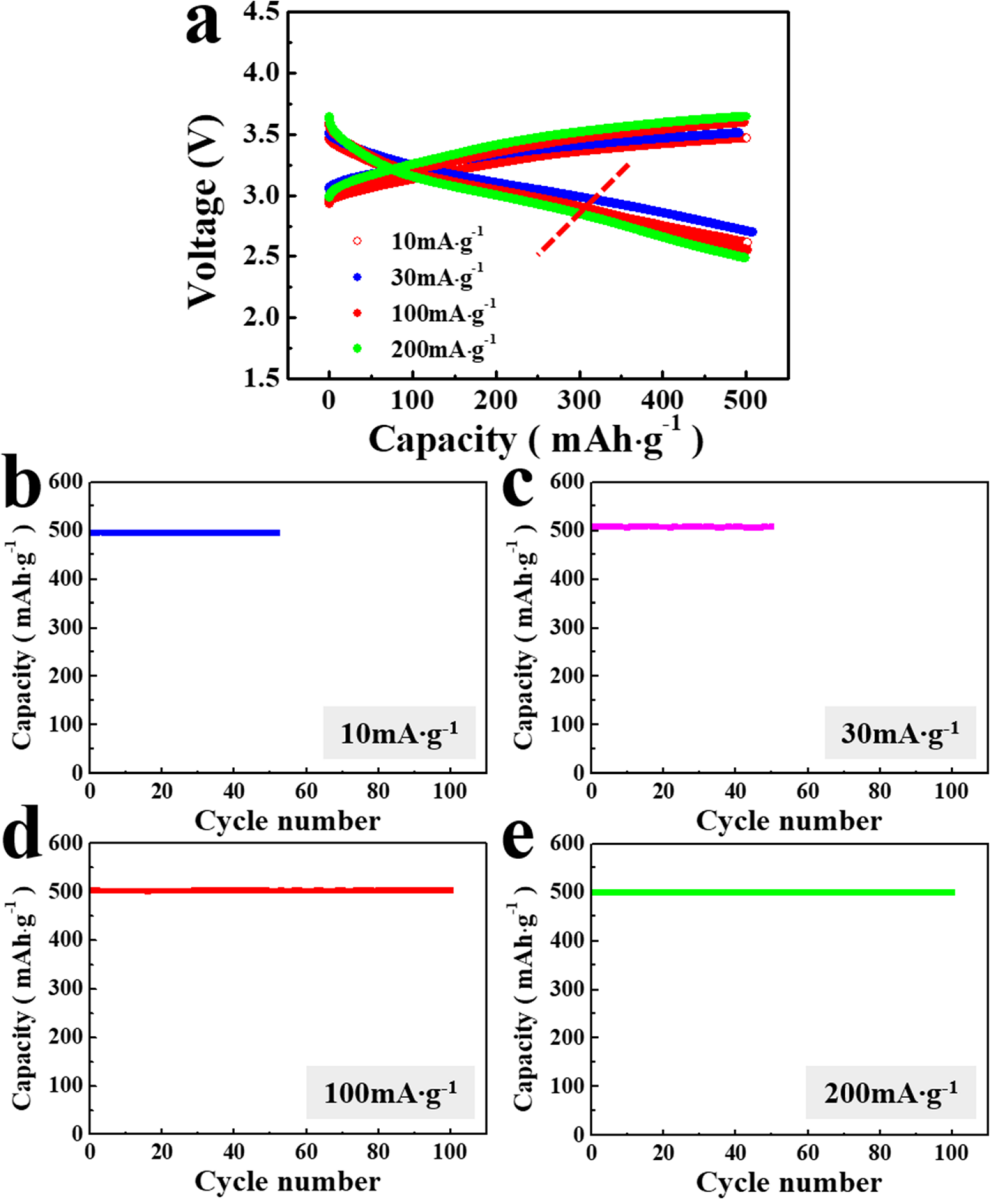
Voltage profile and cyclic performance of the lithia/Li_2_RuO_3_ nanocomposites at current densities of 10, 30, 100, and 200 mA g^−1^ with limited capacity of 500 mAh g^−1^. **a** Voltage profile; cyclic performance at **b** 10 mA g^−1^, **c** 30 mA g^−1^, **d** 100 mA g^−1^, and **e** 200 mA g^−1^

The capacity and cyclic performance of the lithia/Li_2_RuO_3_ nanocomposites were analyzed in more detail using different current densities. Figure 3a compares initial voltage profiles of the nanocomposites at current densities of 10, 30, 100, and 200 mA g^−1^ with a limited capacity of 500 mAh g^−1^. The shape of the voltage profiles does not significantly change as the current density increases. The overpotential of the nanocomposite-containing cell is much lower than that of a typical lithium-air cell, even though both systems are similarly rooted in the ORR. While the lithium-air system is accompanied by a large structural change of the cathode between the gas and condensed phases during charging and discharging, lithia-based cathodes process the redox reaction and retain the condensed phase (solid). This leads to reduction of the energy barrier attributed to electron and ion transfer and phase transformation during charging and discharging, resulting in a relatively low overpotential for the lithia-based system.

**Fig. 4:**
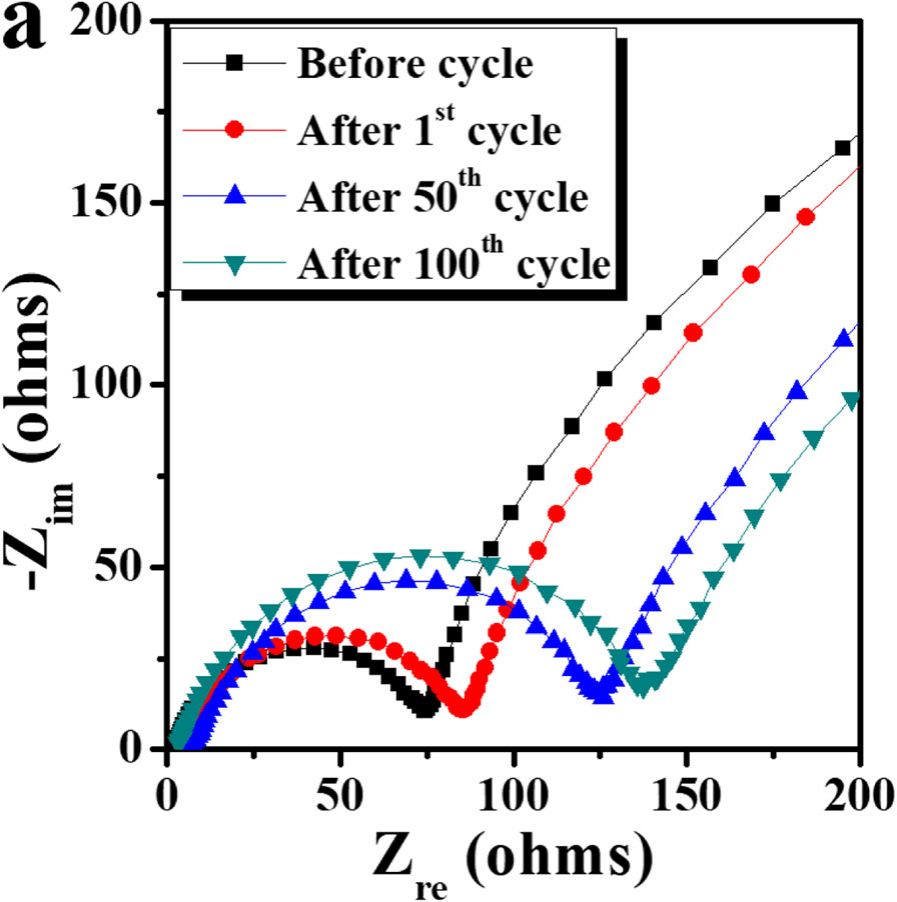
Nyquist plots of the cells containing lithia/Li_2_RuO_3_ nanocomposites before cycling and after selected cycles (1st, 50th, and 100th cycles)

The cyclic performance of the lithia/Li_2_RuO_3_ nanocomposites is stable in the limited capacity of 500 mAh g^−1^ (Fig. 3b–e). Nyquist plots of the cells containing nanocomposites before cycling and after selected cycles (i.e., the 1st, 50th, and 100th cycles) were analyzed to determine the impedance value during cycling. As shown in Fig. 4, the size of the semicircle portion of the Nyquist plot just slightly increases after the 1st cycle compared with that measured before cycling. This indicates that the impedance value, generally attributed to the charge transfer resistance and solid-electrolyte interface, does not significantly change during the initial cycle. After the 50th cycle, the impedance of the cells increases, but the increase in impedance after the 100th cycle is less pronounced, suggesting that the impedance of the cells is relatively stable during cycling, albeit somewhat increased.

**Fig. 5:**
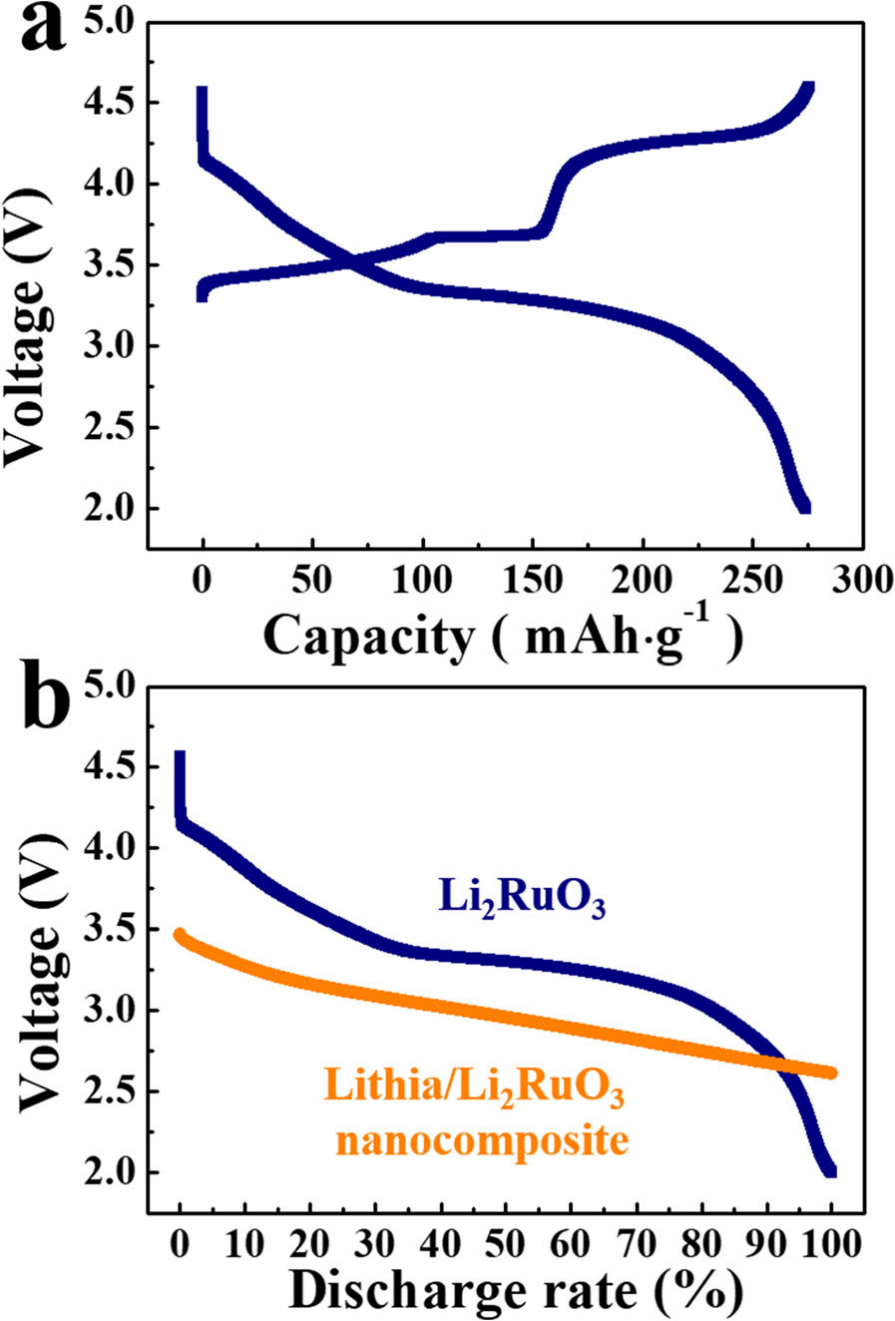
**a **Charge-discharge profile of Li_2_RuO_3_. **b** Comparison of the discharge profile between lithia/Li_2_RuO_3_ nanocomposite and Li_2_RuO_3_

Analysis of the discharging curves of nanocomposites (Fig. 3a) reveals a slight change in the slope near 2.9 to 2.7 V (Fig. 3a, marked with a red line), which appears more clearly when the current density is high. Considering the ORR of lithia, the high voltage region above the red line is associated with annihilation of peroxo-like (O_2_)^*n*−^species formed during the charging process, and the low voltage region is attributed to the neutralization of O 2p holes [27]. It is noteworthy that some of the capacity is observed above ~ 3.1 V (Fig. 3a), since the discharge capacity due to the pure redox reaction of lithia has been shown in the low voltage range < 3.1 V [25–29]. Therefore, the capacity of the lithia/Li_2_RuO_3_ nanocomposites above ~ 3.1 V may be attributed to other materials and not to lithia. Li_2_RuO_3_ contained in the lithia/Li_2_RuO_3_ nanocomposite can contribute to the total capacity because it possesses considerable discharge capacity. To observe the charging-discharging properties of Li_2_RuO_3_, cells containing Li_2_RuO_3_ as the cathode were prepared and the voltage curve was measured. As shown in Fig. 5a, sufficient charging requires a high voltage of ~ 4.3 V, with most of the discharge capacity occurring above 3.1 V. Figure 5b compares the discharge profile of the lithia/Li_2_RuO_3_ nanocomposite and Li_2_RuO_3_, showing that the voltage range of the two cathodes differ. Therefore, the majority of the discharge capacity of the lithia/Li_2_RuO_3_ nanocomposite is related to the redox reaction of the lithia and not to the capacity of Li_2_RuO_3_. However, there is a possibility that some of the capacities of the lithia/Li_2_RuO_3_ nanocomposite observed above ~ 3.1 V is due to the redox reaction of Li_2_RuO_3_, though this is unlikely because a considerable amount of Li_2_RuO_3_ decomposed into Ru during the milling process. Moreover, residual Li_2_RuO_3_ changed to an amorphous phase, as confirmed in Fig. 1a.

**Fig. 6:**
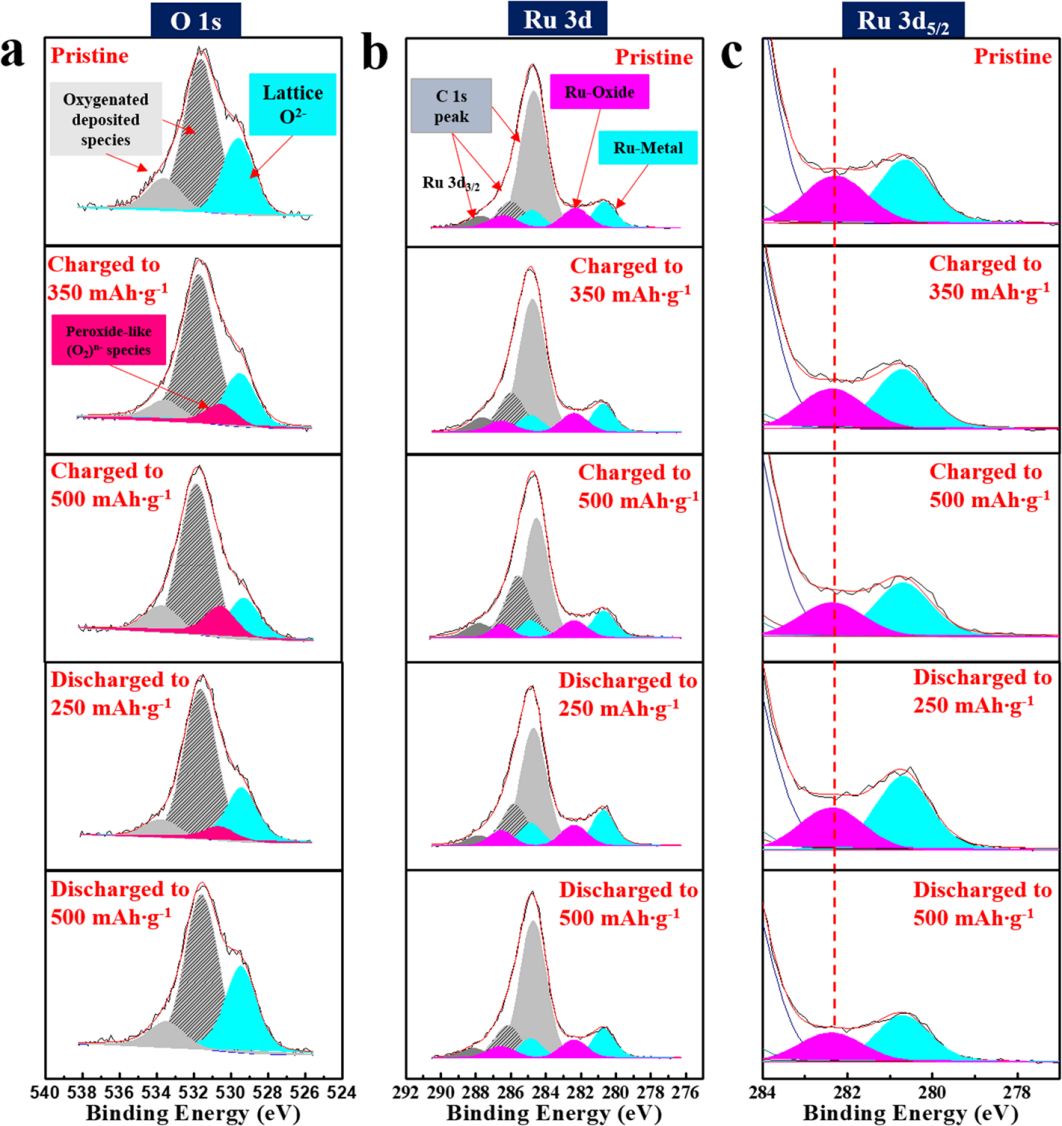
XPS spectra of the lithia/Li_2_RuO_3_ nanocomposites measured at various charging and discharging points. For the measurement, the nanocomposites were charged to 350 and 500 mAh g^−1^ (assigned as full charge), and discharged to 250 and 500 mAh g^−1^ after full charge. **a** O 1 s spectra, **b** Ru 3d spectra, and **c** Ru 3d_5/2_ spectra

To elucidate the redox reaction that occurs during the charging/discharging process, lithia/Li_2_RuO_3_ nanocomposites at different charged and discharged states were analyzed using XPS. Figure 6 shows the O 1 s and Ru 3d spectra of the lithia/Li_2_RuO_3_ nanocomposites during a cycle. For the measurement, the nanocomposites were charged to 350 and 500 mAhg^−1^ (assigned as fully charged), and then discharged to 250 and 500 mAh g^−1^, respectively. As shown in Fig. 6a, the O 1 s spectra of the nanocomposite changed during the charging process. The large peaks at ~ 531.5 eV and ~ 533.5 eV are attributed to oxygenated deposited species from the decomposition of carbonated solvents [39, 40]. When the sample charged to 350 mAh g^−1^, the lattice O^2−^ peak at ~ 529.5 eV (marked in sky blue) decreased and the new peak at ~ 531 eV (marked in red) appeared. The new peak grew as the cell was fully charged to 500 mAh g^−1^, accompanied by a decrease in the lattice O^2−^ peak. This new peak represents the formation of peroxo-like (O_2_)^*n*−^ species through the ORR of lattice oxygen (O^2−^). These species are unstable and readily soluble in the liquid electrolyte; however, the XPS spectra show that they exist in the solid structure, which is likely due to the catalyst. When the sample is discharged, the peaks related to the peroxo-like (O_2_)^*n*−^ species decrease and nearly disappear (when fully discharged to 500 mAh g^−1^), which is accompanied by an increase in the lattice O^2−^ peak. This confirms that the anionic redox reaction by oxygen proceeds reversibly in the lithia/Li_2_RuO_3_ nanocomposites during the charging/discharging process. Figure 6 b shows the Ru 3d spectra, with the Ru 3d_5/2_ section enlarged in Fig. 6c; the Ru 3d peak does not shift markedly during cycling, indicating that the oxidation state of Ru does not change. This is notable because the capacity of Li_2_RuO_3_ attributed to the cationic redox reaction is accompanied by a change in the oxidation state of Ru in addition to the anionic (oxygen) redox reaction. A previous report of the Ru 3d peak shift during cycling was clearly observed through XPS analysis of Li_2_RuO_3_ [40]. However, based on our results, the cationic redox reaction due to Ru barely contributes to the discharge capacity of the lithia/Li_2_RuO_3_ nanocomposites. As discussed previously, it was suspicious that a considerable portion of the capacity of the lithia/Li_2_RuO_3_ nanocomposite comes from the capacity of Li_2_RuO_3_ because Li_2_RuO_3_ in the nanocomposite has the ability to display a large capacity. However, considering the fact that the capacity of Li_2_RuO_3_ is largely attributed to the cationic redox reaction of Ru, it is clear that most of the capacity for the lithia/Li_2_RuO_3_ nanocomposites is generated from the pure oxygen redox of lithia. It is still possible that oxygen in the amorphous Li_2_RuO_3_ structure can also participate in the ORR because the transition from crystalline to amorphous phase could change the electrochemical performance. Moreover, lithium oxide species formed from the decomposition of Li_2_RuO_3_ during milling can have a capacity as well. The small portion of capacity observed above ~ 3.1 V may be related to these additional redox reactions.

## Conclusions

A lithia/Li_2_RuO_3_ nanocomposite was prepared through a milling process, and the structural and electrochemical performance was characterized. Li_2_RuO_3_ was used as a new catalyst for activating lithia and stabilizing unstable reaction products, such as Li_2_O_2_ and LiO_2_. During the milling process, a considerable amount of Li_2_RuO_3_ decomposed to Ru, while what remained transformed to an amorphous phase. Crystalline lithia also changed to an amorphous phase during the milling process. The lithia/Li_2_RuO_3_ nanocomposites show stable cyclic performance until the limited capacity is reached at 500 mAh g^−1^. However, when the limited capacity was increased to 600 mAh g^−1^, cycling resulted in instability, indicating that the cell was overcharged beyond the limit that can be stably charged and discharged. From XPS analysis, it is confirmed that the capacity of the lithia/Li_2_RuO_3_ nanocomposites is mainly attributed to the reversible formation and dissociation of the peroxo-like (O_2_)^*n*−^ species. In contrast, the Ru 3d spectra did not noticeably change during cycling, confirming that the contribution of the cationic (Ru) redox reaction for the capacity of the lithia/Li_2_RuO_3_ nanocomposites is negligible. Therefore, a majority of the capacity of the lithia/Li_2_RuO_3_ nanocomposites is attributed to the oxygen redox of lithia. However, some amount of the capacity, specifically capacity in the high voltage region above ~ 3.1 V, may be related to other material present in the nanocomposites, such as lithium oxide species formed from the decomposition of Li_2_RuO_3_. We believe lithia/Li_2_RuO_3_ nanocomposites can be good candidates for development of lithia-based cathodes with high capacity. It is our hope that this work can contribute to the understanding of lithia/Li_2_RuO_3_ nanocomposites and stimulate the study of lithia-based cathodes.

## Data Availability

Authors declare that the materials, data, and associated protocols are available to the readers, and all the data used for the analysis are included in this article.

## Abbreviations

ORR: Oxygen redox reaction
XRD: X-ray diffractometry
XPS: X-ray photoelectron spectroscopy
EDS: Energy dispersive X-ray spectroscopy
PVDF: Polyvinylidene fluoride
TEM: Transmission electron microscopy
